# A Comparison of Neonatal Outcomes Between Obese and Nonobese Women With Preterm Prelabor Rupture of Membranes

**DOI:** 10.7759/cureus.61754

**Published:** 2024-06-05

**Authors:** Lindsey Robinson, Kiele Reiss, Dara Seybold, Lana Younis, Byron Calhoun

**Affiliations:** 1 Obstetrics and Gynecology, West Virginia University School of Medicine, Morgantown, USA; 2 Obstetrics and Gynecology, West Virginia School of Osteopathic Medicine, Lewisburg, USA; 3 Obstetrics and Gynecology, Institute of Academic Medicine, Charleston Area Medical Center, Charleston, USA; 4 Obstetrics and Gynecology, Charleston Area Medical Center, Charleston, USA; 5 Maternal-Fetal Medicine, West Virginia University, Charleston, USA

**Keywords:** neonatal, intensive care, pregnancy complications, premature rupture, fetal membranes, obesity, body mass index: bmi

## Abstract

Introduction

Preterm prelabor rupture of membrane (PPROM) contributes to increasing rates of preterm birth, causing greater health risks for newborns. While the mechanisms driving PPROM are not well understood, one hypothesis is that it is due to systemic inflammation, which can be caused by obesity defined as a BMI $\geq$30 kg/m^2^. The specific aim of the study was to compare neonatal outcomes after PPROM between patients who were obese vs not obese in early pregnancy at a tertiary medical center serving an Appalachian population.

Methods

An observational, descriptive retrospective review was conducted of the medical records of patients who were diagnosed with PPROM from January 2017 through December 2020. Patients with a single gestation at the time of PPROM without evidence of clinical infection requiring immediate delivery were included. Maternal characteristics, latency management, and birth outcomes were compared between obese ($\geq$30 BMI) and non-obese (<30 BMI) patients.

Results

Of the 214 women in the study, 129 (60.3%) were obese pre-pregnancy and 85 (39.7%) were not. Most PPROM occurred between 32 and 36 weeks of gestation (145 patients, 67.8%), with 19.2% occurring at 26-31 weeks (41 patients), and 13.2% at <26 weeks of gestation (28 patients). Latency, defined as the days between PPROM and delivery, ranged from 0 to 80 days with a mean of 4.9 + 10.9 days. At least one day of latency was achieved for most patients (144/214; 67.3%). When outcomes were compared between obese and nonobese patients, the obese patients experienced significantly more complications (10.1% vs 2.4%; p=0.031), which were accompanied by greater neonatal morbidity 67 of 129 ((51.9%) vs 30 of 85 (35.3%); p=0.018). Obese women had greater odds that their newborns would experience neonatal morbidity than nonobese women (odds ratio, 1.98; 95% confidence interval, 1.1-3.5).

Conclusion

This study of Appalachian women found that pre-pregnancy BMI $\geq$30 increased the risk of complications and neonatal morbidity after PPROM. To improve birth outcomes, healthcare workers and policymakers must work together to decrease rates of obesity in Appalachian women at or near childbearing age.

## Introduction

Obesity (defined in terms of body mass index (BMI) ≥30) or overweight (BMI 25 to <30) during pregnancy has been estimated to contribute to 25% of pregnancy complications [1]. One such complication is preterm prelabor rupture of membrane (PPROM). PPROM is defined as the rupture of amniotic membranes before 37 weeks gestation of pregnancy. Up to 3% of pregnancies are complicated by PPROM [2]. This rupture increases the risk of infection and preterm birth, which is the most common cause of neonatal death and morbidity in the world [3]. Specifically, obesity in pregnancy has been associated with an increased risk for PPROM [4]. It was found that PPROM was increased in patients with body mass index (BMI) >25 in a study of 3,198 nulliparous women in a homogeneous Chinese population [5]. In a 2019 study, there was an increased risk of PPROM with preterm delivery in patients with BMI >25 in a heterogenous Brazilian population of 4,150 women [6]. While the mechanism of this is not well understood, one hypothesis is that it is due to the systemic inflammation caused by obesity [7,8].

Obesity has been described as an upregulated inflammatory state with proinflammatory cytokines, adipokines, and changes in the hypothalamic-pituitary-adrenal axis with the release of corticotropin-releasing factor. Excess adipose tissue and nutrients cause increased secretion of tumor necrosis factor-alpha (TNF-α) and interleukin 6 leading to increased C-reactive protein (CRP) and overall inflammation [9]. All of these create an environment for inflammatory changes in the fetal membranes and the induction of proteases that weaken the amniotic membranes and contribute to PPROM. There is recent evidence that prepregnancy obesity is associated with higher rates of PPROM [8]. Obesity has also been correlated with an increase in adverse neonatal outcomes, which include respiratory morbidity, infections, and all-cause neonatal mortality [5,8].

The literature remains unclear, however, regarding the association between increased BMI and complications after PPROM. In a 2020 study of 331 American women with PPROM, no difference in the duration/latency of PPROM to delivery in patients with a BMI ≥30 as compared with women with a BMI <30 was found [10]. Our study’s primary aim was to compare neonatal outcomes between obese and nonobese patients with PPROM cared for at a tertiary medical center serving an Appalachian population.

## Materials and methods

Study design

An observational, descriptive research design was employed using a retrospective review of the medical records of women who were diagnosed with PPROM using International Classification of Diseases (ICD) code O42.113 during prenatal care at a single tertiary medical center. Study variables were obtained through our electronic data warehouse retrieval. Variables that could not be obtained by this method were then abstracted from electronic medical records.

Selection criteria

All patients who experienced PPROM between January 9, 2017 and December 28, 2020 with a living fetus (ICD Z37.0) without evidence of clinical infection requiring immediate delivery (ICD O42.113) were included. Infection was defined as a maternal fever (ICD O75.2), uterine tenderness (ICD R10.2), and contractions at the time of PPROM precluding conservative management and requiring immediate delivery. In addition, the following were also excluded: lethal fetal anomalies (ICD O35.8XX0), anencephaly (ICD Q00.0), renal agenesis (ICD Q60.0), and serious fetal cardiac anomalies including hypoplastic left heart (ICD Q23.4), transposition of great vessels (ICD Q20.3), conotruncal defects (ICD Q24.9), and univentricular hearts (ICD Q20.4). PPROM management included aggressive use of antepartum steroids, fetal monitoring, and latency antibiotics.

Clinical data collection

The following patient characteristics were collected from the medical record: maternal age, gravidity, parity, diabetes, prepregnancy BMI, hypertension, history of preterm birth, thyroid disease, thrombophilia, tobacco use, alcohol use, and substance use. Prepregnancy BMI was reported from the self-reported height and weight provided by the patient during the initial prenatal visit intake. BMI was calculated as weight (kg) divided by height (m) squared. A BMI of ≥30 kg/m^2^ was considered obese. If a prepregnancy BMI was not in the patient’s medical record, the BMI from the patient’s initial prenatal visit was used. Pregnancy data collected included weeks gestation at PPROM and latency. Latency was defined as the interval between rupture of membranes (the amniotic sac) and the onset of labor. We assessed the following outcomes: type of delivery, gestational age at birth, maternal morbidity, neonatal death, neonatal intensive care unit (NICU) admissions, and neonatal morbidity. Neonatal morbidity was defined as the risk of death in first 28 days of life. For purposes of this study, if one or more medical conditions was present that increased risk of death, then the newborn had a neonatal morbidity. Medical conditions were recorded and reported for each patient. Neonatal morbidity included infections, lung problems, intraventricular hemorrhage, and other conditions. Maternal morbidity included infection, perinatal complications, a need for blood products, and postpartum readmission.

Statistical analysis

Data were analyzed using IBM Corp (Released 2010; IBM SPSS Statistics for Windows, Version 19.0; Armonk NY: IBM Corp). To answer the research question of whether birth outcomes are associated with prepregnancy BMI ≥30 kg/m^2^, the study population was grouped into BMI ≥30 kg/m^2^ and <30 kg/m^2^. Maternal and pregnancy characteristics and maternal and neonate outcomes were compared between these two BMI study groups. Latency as a categorical variable of at least one day and as a continuous variable (number of days) was compared across gestational age groups. Continuous variables are presented as mean and standard deviation (SD) and were compared using student independent-sample t-tests, while categorical variables are reported as percentages and were compared using chi-squared or Fisher’s exact test to determine if there was an association between a BMI ≥30 kg/m^2 ^and neonatal outcomes. Odds ratios (ORs) and 95% confidence intervals (CIs) were calculated for birth outcomes to determine the odds of prepregnancy BMI ≥30 kg/m^2 ^compared with <30 kg/m^2^. An alpha of 0.05 was set in determination of statistical significance. Data were reported in tables and narration in the results section.

## Results

Of the 253 women with PPROM during the study period, 22 were excluded because they were twin gestations, and 17 were excluded due to no delivery data at the study tertiary medical center, providing a final study sample of 214 women. The prepregnancy obesity rate in our study population was 60.3%, dividing our study group into 129 women in the obese group (BMI >30) and 85 women in the nonobese group (BMI ≤30; 39.7%). The obese women in our cohort were more likely to have diabetes than the women who were not obese (18.6% vs 4.7%, p=0.003), and the women who were not obese were more likely to use tetrahydrocannabinol (THC)(31.8% vs18.8%, p=0.033) (Table 1).

**Table 1:** Maternal and Pregnancy Characteristics. BMI, body mass index; GDM, gestational diabetes; PPROM, preterm prelabor rupture of membranes; THC, tetrahydrocannabinol. ^a^Data are given as n (%) unless indicated otherwise. ^b^Mean + SD.

Characteristic^a^	All patients	Prepregnancy BMI		P value
	n=214	≥30 (n=129)	< 30 (n=85)	
Maternal Age (years)^b^	27.2+5.9	27.3+6.0	27.1 +5.8	0.904
Parity				0.773
Nulliparous	79 (36.9)	49 (38.0)	30 (35.3)	
Multiparous	135 (63.1)	80 (62.0)	55 (64.7)	
Gravidity^b^	2.7+1.7	2.8+1.5^ b^	2.8+1.9	0.424
Diabetes	28 (13.1)	24 (18.6)	6 (7.1)	0.003
GDM	20 (9.3)	16 (12.4)	4 (4.7)	0.018
Hypertension	34 (15.9)	25 (19.4)	9 (10.6)	0.090
History of preterm birth	52 (24.3)	28 (21.7)	24 (28.2)	0.329
Thyroid disease	6 (2.8)	4 (3.1)	2 (2.4)	1.000
Thrombophilia	4 (1.9)	2 (1.6)	2 (2.4)	0.650
Tobacco use	94 (43.9)	48 (37.2)	46 (54.1)	0.017
Number of cigarettes per day^b^	12.0+6.6	12.05+6.8	11.92+6.5	0.933
Alcohol use	2 (0.9)	2 (1.6)	0 (0.0)	0.317
Substance use	91 (42.5)	48 (37.2)	43 (50.6)	0.066
Opioid	28 (13.1)	12 (9.3)	16 (18.8)	0.061
THC	51 (23.8)	24 (18.8)	27 (31.8)	0.033
Weeks gestation at PPROM^b^	31.6+4.8	31.8+4.6	31.4+5.0	0.544
Latency (one or more days)	144 (67.3)	81 (62.8)	63 (74.1)	0.102
Latency (time to delivery in days)^b^	4.8+10.9	5.1+11.4	4.4+10.1	0.616

Most PPROM occurred between 32 and 36 weeks of gestation (145 patients, 67.8%), with 19.2% occurring at 26-31 weeks (41 patients), and 13.2% at <26 weeks of gestation (28 patients). Latency, defined as the days between PPROM and delivery, ranged from 0 to 80 days with a mean of 4.9+10.9 days. At least one day of latency was achieved for most patients (144 of 214, 67.3%). There were no differences in when PPROM occurred during gestation or in latency between the obese and nonobese patients (Table 2).

**Table 2:** Latency by Weeks Gestation at PPROM. PPROM, preterm prelabor rupture of membranes. ^a^Data are given as n (%) unless indicated otherwise. ^b^Mean+SD.

Gestational age group^a^	All patients with PPROM	Latency		Length of latency, days^b ^
	(n=214)	Yes (n=144)	No (n=70)	(n=144)
<26 weeks	28 (13.1)	22 (15.3)	6 (8.6)	17.7+21.6
26-31 weeks	41 (19.2)	36 (25.0)	5 (7.1)	10.6+14.7
32-34 weeks	70 (32.7)	51 (35.4)	19 (27.1)	4.0+4.1
35-36 weeks	75 (35.0)	35 (24.3)	40 (57.1)	1.5+1.9

Approximately half (50.5%) of the patients in the study experienced at least one maternal morbidity. When outcomes were compared between obese and nonobese patients, obese patients experienced significantly more perinatal complications (10.1% vs 2.4%; p=0.031). Perinatal complications included postpartum bleeding/hemorrhage, placental abruption, retained placenta, preeclampsia, breech delivery, and prolapsed umbilical cord. While 98 (45.8%) study patients had infections at delivery, there was no difference between the obese and nonobese patients (p=0.718). Vaginal infections were the leading type of infection (76 patients; 35.5%) with the obese patients having higher rates compared with the nonobese patients (20.2% vs 8.2%, p=0.020) (Table 3).

**Table 3:** Maternal Outcomes. BMI, body mass index; BV, bacterial vaginosis; GBS, group B Streptococcus; HCV, hepatitis C; STD, sexually transmitted disease; UTI, urinary tract infection. ^a^Data are given as n (%) unless indicated otherwise. ^b^Mean+SD.

Outcome^a^	All patients	Prepregnancy BMI		P value
	n=214	≥30 (n=129)	<30 (n=85)	
Delivery				
Cesarean	83 (38.8)	54 (41.9)	29 (34.1)	0.316
Vaginal	131 (61.2)	75 (58.1)	56 (65.9)	
Spontaneous	128 (59.8)	75 (58.1)	53 (62.4)	0.316
Induced	23 (10.7)	14 (10.9)	9 (10.6)	1.000
Assisted (forceps, vacuum)	6 (2.8)	4 (3.1)	4 (4.7)	0.716
Gestational age at birth (weeks)^b^	32.2+4.6	32.4+4.3	31.9+5.0	0.394
Maternal morbidity	108 (50.5)	68 (52.7)	40 (47.1)	0.485
Perinatal complication	15 (7.0)	13 (10.1)	2 (2.4)	0.031
Need for blood products	5 (2.3)	3 (2.3)	2 (2.4)	0.718
Postpartum readmission	3 (1.4)	2 (1.6)	1 (1.2)	0.933
Infection at delivery	98 (45.8)	58 (45.0)	40 (47.1)	0.781
HCV	23 (10.7)	12 (9.3)	11 (12.9)	0.359
Chorioamnionitis	14 (6.5)	8 (6.2)	6 (7.1)	0.786
UTI	7 (3.3)	5 (3.9)	2 (2.4)	0.706
Vaginal	76 (35.5)	47 (36.4)	29 (34.1)	0.772
STD	17 (7.9)	8 (6.2)	9 (10.6)	0.418
BV	44 (20.6)	24 (18.6)	20 (23.5)	0.393
Candida	6 (15.4)	3 (2.3)	3 (3.5)	1.000
GBS	33 (15.4)	26 (20.2)	7 (8.2)	0.020

Obese patients experienced greater neonatal morbidity (67 of 129 (51.9%) vs 30 of 85 (35.3%); p=0.018). Respiratory distress syndrome rates were the highest at 31.8% of neonatal morbidity. Obese women had greater odds that their newborns would experience neonatal morbidity than nonobese women (odds ratio, 1.98; 95% confidence interval, 1.1-3.5) (Table 4).

**Table 4:** Neonatal Outcomes. BMI, body mass index; BPD, bronchopulmonary dysplasia; CL, confidence intervals; IVF, intraventricular hemorrhage; LFT, liver function test; NICU, neonatal intensive care unit; OR, odds ratio; RDS, respiratory distress syndrome; ROP, retinopathy of prematurity. ^a^Data are given as n (%) unless indicated otherwise. ^b^Mean+SD.

Outcome^a^	All patients (n=214)	Prepregnancy BMI		OR (95% CI)	P value
		≥30 (n=129)	<30 (n=85)		
Neonatal death	23 (10.7)	12 (9.3)	11 (12.9)	0.69 (0.29-1.64)	0.499
NICU admissions	79 (36.9)	48 (37.2)	31 (36.5)	1.03 (0.59-1.82)	1.000
Days in NICU^b^	11.8+20.5	13.5+23.5	9.5 +15.9		0.237
Neonatal morbidity	97 (45.3)	67 (51.9)	30 (35.3)	1.98 (1.13-3.48)	0.018
RDS	69 (31.8)	43 (33.3)	25 (29.4)	1.20 (0.66-2.17)	0.327
Sepsis	14 (6.5)	10 (7.8)	4 (4.7)	1.70 (0.52-5.61)	0.533
Patent ductus arteriosus	13 (6.1)	11 (8.5)	2 (2.4)	3.87 (0.84-17.91)	0.081
BPD	10 (4.7)	8 (6.2)	2 (2.4)	2.74 (0.57-13.25)	0.389
IVF	8 (3.7)	5 (3.9)	3 (3.5)	1.10 (0.26-4.74)	0.737
Severe ROP	4 (1.9)	4 (3.1)	0 (0.0)	0.60 (0.53-0.67)	0.153
Pulmonary hypertension	4 (1.9)	3 (2.3)	1 (1.2)	2.00 (0.21-19.55)	1.000
Necrotizing enterocolitis	0 (0.0)	0 (0.0)	0 (0.0)		
Ventilator	42 (19.6)	28 (21.7)	14 (16.5)	1.41 (0.69-2.86)	0.383
Conventional	25 (11.7)	19 (14.7)	6 (7.1)	2.27 (0.87-5.95)	0.127
High frequency	5 (2.3)	2 (1.6)	3 (3.5)	0.43 (0.07-2.63)	0.388
Days supported^b^	2.3+8.5	1.8+7.8	1.2+5.9		0.511
Inhaled nitrous oxide	4 (1.9)	4 (3.1)	0 (0.0)	0.60 (0.53-0.67)	0.153
Discharged on oxygen	4 (1.9)	3 (2.3)	1 (1.2)	2.00 (0.21-19.55)	1.000
Abnormal LFT	20 (9.3)	17 (13.2)	3 (3.5)	4.15 (1.18-14.63)	0.017

## Discussion

Our study found that a BMI ≥30 prepregnancy led to an increased risk of perinatal maternal complications and neonatal morbidity after PPROM. Of the patients in this cohort, 60.3% met the criteria for obesity prepregnancy, while only 40% of women of childbearing age in West Virginia are obese [11]. This is indicative of the increased incidence of PPROM in obese women as compared with nonobese women reported by others [4-8]. Patients meeting the criteria for obesity prepregnancy had a significantly higher incidence of diabetes, both gestational diabetes (GDM) and type 2 diabetes.

Prior studies have illustrated the relationship between diabetes and PPROM, which is likely due to an overall elevated inflammatory state of the mother [12]. Preconception counseling and early diagnosis of diabetes can allow women to have better control of blood sugar levels throughout pregnancy. GDM is typically tested by 28 weeks of gestation, which is after some women may already have PPROM. In women with high-risk factors, early glucose tolerance testing should be routinely encouraged as proper diagnosis and treatment of diabetes may decrease the incidence of PPROM and its associated risks. Early treatment of GDM improves maternal and neonatal outcomes [13]. Given this, we suspect that early screening in at-risk populations could decrease the incidence of PPROM and improve outcomes. Further, promoting reproductive health education may help young women to take control over their reproductive health and participate in preconception counseling. To reduce childhood and adolescent obesity which can lead to adult obesity, healthy habits can be promoted in public schools in Appalachia [14].

Although there were more women meeting the criteria for obesity in this cohort of patients than in the general childbearing population, the course of PPROM was relatively similar, regardless of the mother’s BMI. Average latency and gestational age at delivery were not statistically different between groups. Additionally, the patients with elevated BMI did not undergo significantly more cesarean sections in this cohort. Despite this, patients with higher BMI had significantly more perinatal complications and their newborns had more neonatal morbidity than patients with lower BMI.

Our study contributes further information validating the link between obesity and increased risk of PPROM with adverse maternal and neonatal consequences. Although several studies have illustrated a link between increased BMI and perinatal complications, there is conflicting evidence regarding the correlation between obesity and neonatal outcomes when controlled for gestational age at delivery [7,8]. Our study further illustrates the link between obesity and poor neonatal outcomes, likely due to systemic inflammation, given that the gestational age at delivery and latency after PPROM onset were similar between the two groups.

There were limitations and strengths to our study. Limitations of our study include its small size, relatively homogeneous population in Appalachia, and significant maternal tobacco and substance use. Most of the pre-pregnancy BMI was self-reported at initial prenatal visit and if the patient did not know this information, the BMI was calculated from the initial prenatal visit height and weight measurements. The nature of self-reporting may affect accuracy as social desirability bias and recall bias may have been introduced. The single-institution study design and inclusion of all patients admitted to our regional hospital was a strength that facilitated complete records of the patients’ care during their hospital stays and standardization of care for PPROM.

## Conclusions

This study of Appalachian women found that early pregnancy BMI ≥30 increased the risk of complications and neonatal morbidity after PPROM. Our study stresses the need for further delineation of the etiology of PPROM in obese patients and the development of strategies for prevention. Healthy habits in childhood and adolescence could be promoted in public schools in Appalachia in efforts to reduce childhood obesity. Also, education surrounding reproductive health is essential for young women to encourage control over their reproductive health and enable them to optimize their health and undergo adequate preconception counseling before becoming pregnant. For women with BMI ≥30 who become pregnant, providers should aim for early identification and intervention of underlying comorbidities. Healthcare workers and policymakers must work together to decrease rates of obesity in Appalachian women at or near childbearing age.
